# Rapid screening of high expressing *Escherichia coli* colonies using a novel dicistronic-autoinducible system

**DOI:** 10.1186/s12934-021-01711-2

**Published:** 2021-12-11

**Authors:** Fatemeh Sadat Shariati, Dariush Norouzian, Vahideh Valizadeh, Reza Ahangari Cohan, Malihe Keramati

**Affiliations:** Department of Nanobiotechnology, New Technologies Research Group, Pasteur Institute of Iran, Tehran, Iran

**Keywords:** Dicistronic expression system, Rapid screening, *Escherichia coli*, Self-inducible expression system, Enhanced green fluorescent protein

## Abstract

**Background:**

Identification of high-expressing colonies is one of the main concerns in the upstream process of recombinant protein development. The common method to screen high-producing colonies is SDS-PAGE, a laborious and time-consuming process, which is based on a random and qualitative way. The current study describes the design and development of a rapid screening system composed of a dicistronic expression system containing a reporter (enhanced green fluorescent protein, eGFP), protein model (staphylokinase, SAK), and a self-inducible system containing heat shock protein 27 (Hsp27).

**Results:**

Dicistronic-autoinducible system expressed eGFP and SAK successfully in 5-ml and 1-L culture volumes. High expressing colonies were identified during 6 h via fluorescent signals. In addition, the biological activity of the protein model was confirmed semi-quantitatively and quantitatively through radial caseinolytic and chromogenic methods, respectively. There was a direct correlation between eGFP fluorescent intensity and SAK activity. The correlation and linearity of expression between the two genes were respectively confirmed with Pearson correlation and linear regression. Additionally, the precision, limit of detection (LOD), and limit of quantification (LOQ) were determined. The expression of eGFP and SAK was stable during four freeze–thaw cycles. In addition, the developed protocol showed that the transformants can be inoculated directly to the culture, saving time and reducing the error-prone step of colony picking.

**Conclusion:**

The developed system is applicable for rapid screening of high-expressing colonies in most research laboratories. This system can be investigated for other recombinant proteins expressed in *E. coli* with a potential capability for automation and use at larger scales.

**Supplementary Information:**

The online version contains supplementary material available at 10.1186/s12934-021-01711-2.

## Introduction

*Escherichia coli* is a suitable host for the initial screening of recombinant protein expression owing to easy genetic manipulation, inexpensive culture, and rapid growth [1, 2]. However, conventional methods for identification of colonies with high production levels of target protein require several time-consuming steps, including (i) vector transformation, (ii) plating the transformed cells, (iii) colony picking, (iv) inoculating the colony to a preculture, (v) inoculating the final culture with the pre-culture, (vi) optical density monitoring, (vii) induction of expression, and finally, (viii) the investigation of protein expression through a detection method. In this regard, the most common detection method is the SDS-PAGE technique in which several limited clones are randomly analyzed qualitatively or semi-quantitatively to distinguish non- or low-expressing clones from high-expressing ones [2]. The screening with the SDS-PAGE technique is labor-intensive, time-consuming, and usually restricted to a limited number of colons that may lead to missing high expressing colons. The emerging techniques, such as fluorescence-activated cell sorting (FACS) and robotic platforms, facilitate the colony selection program. These systems take the benefit of simultaneous cell sorting based on several parameters and automated systematic screening of multi-well plates [3]. Meanwhile, expensive equipment and special expertise might not be available in every research laboratory. In addition, in these platforms, even with automation, the protein production pipeline often consists of several steps that have to be linked together and require manual interventions. Moreover, these platforms require high-resolution imaging systems to monitor the colonies growing on the agar surface and discriminate them based on user-selectable criteria, such as diameter and roundness. Therefore, these platforms are not cost-effective and in the practice, the screening of expression is carried out semi-quantitatively [4].

Reporter proteins that can fuse to the N- or C-terminus of target proteins have been suggested for the screening of expression [5, 6]. The development of enhanced green fluorescent protein (eGFP) and other fluorescence-based proteins has opened new opportunities for screening purposes. These protein reporters do not need an external substrate or cofactor, work in a wide range of conditions (pH, temperature, salt concentration, detergents, and protease), are non-toxic, and are detectable quantitatively by fluorimetry [4]. However, removal of the reporter is usually necessary following expression since it may affect the structure and function of the target protein [7]. Development and use of dicistronic expression systems have resolved this obstacle [8, 9].

Dicistronic expression systems are classified into two categories. In the first category, dicistronic expression vectors consist of two gene insertion sites in which the expression of each gene was separately controlled under a unique promoter. These systems have a wide application in the coexpression of binary protein complexes or two-independent proteins [9, 10]. However, in these systems, the level of expression in the first position is not proportional to the second position and they therefore cannot be ideal for screening purposes. While in the second category, both genes are controlled under the same promoter but distinct in the ribosome-binding sites (RBS). In these systems, the expression of the second gene is comparable to the first gene and therefore, they are suitable for screening purposes by employing reporters [10–12].

The other strategy to facilitate colony screening is the use of self-inducible expression (SILEX) systems. SILEX systems are efficient producing systems for removing the toxicity effect of inducer on the host cells. These systems do not require optical density monitoring for induction and work at different temperatures (20 to 37 °C) and in a variety of common culture media (LB, TB, and 2YT). They are simpler and more cost-effective than other strategies, such as the use of Studier self-induction culture media or the use of promoters inducing a metabolic change. It is also possible to study the protein expression in 96 well microplates through these systems [13]. Miniaturization of such systems usually saves time and cost and decreases manual handling steps and errors [2, 14].

In the current study, we developed a simple screening system at a micro-scale that can be used for rapid screening of high-expressing colonies in *E. coli*. The designed system is a combination of a dicistronic expression cassette containing T7-promoter, the gene of interest (Staphylokinase, SAK), ribosome binding site, and an eGFP (enhanced green fluorescent protein) as a reporter gene. The pelB sequence was embedded in all constructs for secreting proteins into periplasm to simplify the screening procedure and improve the protein folding. The expression is auto-induced via a self-inducible expression system using heat shock protein 27 (Hsp27).

## Results

### Design of screening protocol

To screen quantitatively high-expressing colonies in a short time, a dicistronic expression system was designed. At this dicistronic system, SAK was selected as a suitable model protein at the first gene site since its activity can be measured quantitatively and qualitatively using chromogenic and caseinolytic assays, respectively. Moreover, an eGFP was utilized as a reporter protein at the second gene site. This system auto-induces the expression of a model protein (SAK) with the leaky expression of Hsp27 and omits the optical density monitoring and the need for an inducer (IPTG). Of course, the proposed protocol can also be investigated for other recombinant proteins.

To set up the protocol, the following several optimization steps were carried out. In the double transformation process, different amounts of plasmids were tested to get approximately 100 double-transformed clones by plating on the LB agar plates containing both selective antibiotics followed by colony counting. The result indicated that 200–300 ng of plasmids are needed to obtain the specified number of double-transformed clones (Additional file 1: Table S1). Moreover, for removing the spreading step of newly double transformed cells on LB agar plate, direct inoculation of transformation solution to each well of 96-microplate was proposed. For this reason, the inoculation was examined at different volumes of the newly transformed suspension on LB agar plates containing both selective antibiotics to obtain a single clone in each well of 96-microplate. The data revealed that 10 µl is the smallest volume of the newly transformed bacterial suspension (according to facilitated screening protocol) to obtain a single clone (Additional file 2: Table S2). Elimination of colony culturing step on LB agar plate and the removal of preculture step reduces the time of screening process by up to 2 days compared to the SDS-PAGE technique.

The optimum time for fluorimetry was achieved at 6 h after the inoculation of transformants. As depicted in Additional file 3: Fig. S1, the slope of the graph is higher at 6 h than the other times. Therefore, eGFP expression level among different clones is determined quantitatively 6 h after inoculation via fluorescent signals while in the SDS-PAGE technique, the expression results of different clones are usually investigated qualitatively or semi-quantitatively at 6 to 18 h after inoculation. The fluorescence sensitivity of the fluorimeter was set to 60 RFU (Relative Fluorescent Unit) to obtain a reliable response (Additional file 4: Fig. S2). The fluorescence signal measurements and SAK activity assay were performed to check the correlation between the model protein and eGFP expressions in the first and second insertion sites, respectively. In addition, the optimization of double-transformation for obtaining ~ 100 clones and the step to obtain a double transformed clone in each well of 96-microplate was repeated three times in three different days (with a one-week interval) to check the precision, linearity, limit of detection (LOD), and limit of quantification (LOQ) (Table 1) [15]. The results revealed a suitable precision for all the steps in the proposed protocol. The linearity between the fluorescence signal and the SAK activity was confirmed through the linear regression method with a coefficient of determination (R^2^) of 0.9623. Thus, by measuring fluorescent signals in different clones of the designed system, high-staphylokinase expressing clones were screened in a short time. The LOD and LOQ for fluorimetry signals were respectively calculated as 3155.52 (Mean + 3SD) and 3497.40 (Mean + 10SD) RFU. These values were corresponded to 0.68 IU/mg and 27.53 IU/mg of SAK activity, respectively. In conventional screening techniques, such as SDS-PAGE, the sensitivity of detection mainly depends on the gel staining method, for example 30 ng for Colloidal Coomassie Brilliant Blue G-250, 100 ng for Coomassie Brilliant Blue R-250, and ~ 5–10 ng for silver staining. Although silver staining has a high sensitivity for detecting purified proteins at nano-gram range, this method can only qualitatively detect the expression of target protein in the cell extract. While the proposed protocol can measure the smaller amount of target protein in the cell extract via fluorescent signals.

**Table 1:** Calculation of validation parameters for the screening protocol

Step	Average	SD	RSD (%)	LOD	LOQ
Fluorescence signal measurement (RFU)	34,791.44	± 753.31	2.16	3155.52	3497.40
SAK activity assay (IU/mg)	184.87	± 3.18	1.72	0.68	27.53
Number of double transformed clones	107.33	± 2.5	2.32	–	–
Obtaining a clone in each well of 96-microplate	1	± 0.00	0	–	–

SD: standard deviation; RSD: relative standard deviation; LOD: limit of detection; LOQ: limit of quantification

### Expression analysis of SAK with SDS-PAGE, western blot, and purification

**Fig. 1:**
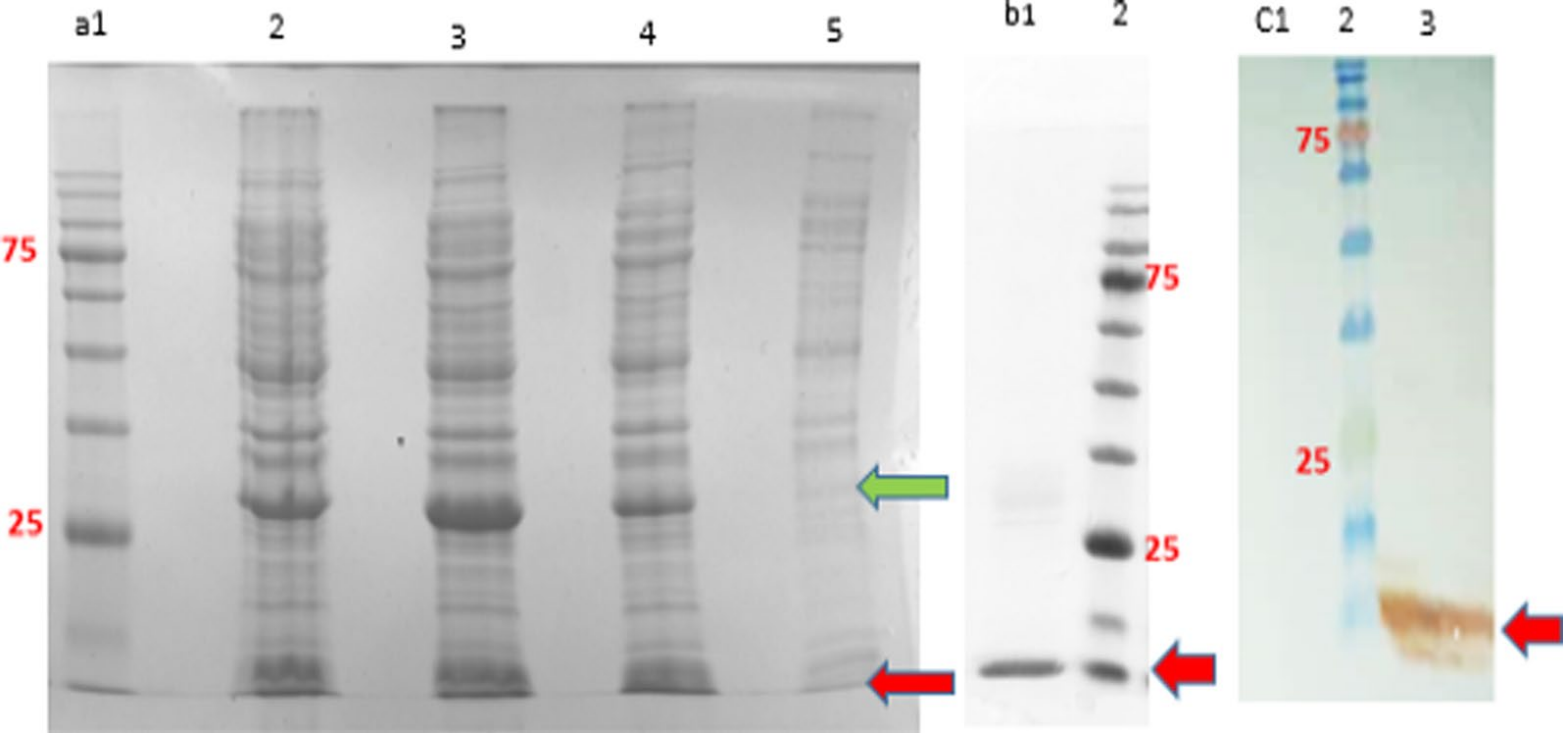
The expression analysis of eGFP and SAK proteins in dicistronic SILEX system. **a** 15% SDS-PAGE analysis of eGFP and SAK expression [Lane 1: protein marker, Lanes 2 and 3: 16 h after inoculation in 1-L culture at two different clones, lane 4: 16 h after inoculation in 5-ml culture, and Lane 5: 2 h after inoculation at 5-ml culture], **b** 15% SDS-PAGE analysis of purified SAK [Lane1: purified SAK, Lane 2: protein marker], **c** western blot analysis of SAK protein [Lane 1: *E. coli* BL21 (DE3) containing pET28a-*sak-rbs*-*egfp* (negative control), Lane 2: protein marker, Lane 3: double transformed *E. coli* BL21 (DE3). The green and red arrows indicate eGFP and SAK proteins, respectively. The protein marker molecular weights are 180, 135, 100, 75, 63, 48, 35, 25, 17, and 11 kDa

The expression of eGFP and SAK proteins through the Hsp27 SILEX system was analyzed applying 15% SDS-PAGE at 5-ml and 1-L cultures. The stained gels showed that eGFP and SAK proteins were expressed respectively at molecular weights of ~ 28 and 15.5 kDa, (Fig. 1a). Moreover, the SAK expression was also confirmed after affinity purification (Fig. 1b) and western blot (Fig. 1c).

### Enzyme activity measurements

Radial caseinolytic and chromogenic methods were employed to confirm the activity of SAK after expression in dicistronic SILEX system. A clear zone was detected around the wells on account of the proteolytic activity of the enzyme in caseinolytic assay (Additional file 5: Fig. S3). The SAK activity measured with radial caseinolytic assay for three different clones is summarized in Table 2. The activity of expressed SAK in the selected clones was also confirmed through the chromogenic method (Table 3). The results shed light on the linear relationship between the SAK activity (IU/mg) and the absorbance at a wavelength of 405 nm (Additional file 6: Fig. S4).

**Table 2:** The result of radial caseinolytic assay of SAK

Sample	Clone 1	Clone 2	Clone 3	Positive Control^a^	Negative Control^b^
Clear zone (cm)	1.47 ± 0.02	1.44 ± 0.03	1.44 ± 0.02	1.53 ± 0.03	0.61 ± 0.01
RSD (%)	1.31	2.30	1.50	2.43	2.67

RSD: Relative standard deviation

^a^Recombinant SAK with plasminogen

^b^Recombinant SAK without plasminogen. Data are represented as Mean ± SD from three independent experiments

**Table 3:** The enzyme activity measurement using chromogenic method

Sample	Clone 1	Clone 2	Clone 3	Positive Control^a^	Negative Control^b^
SAK activity (IU/mg)	188.32 ± 2.18	171.29 ± 6.26	143.59 ± 5.11	183.37 ± 4.29	0
RSD (%)	1.15	3.65	3.56	2.34	0

RSD: Relative standard deviation

^a^Recombinant SAK with plasminogen

^b^Recombinant SAK without plasminogen. Data are represented as Mean ± SD from three independent experiments

### Correlation between fluorescence signals and SAK chromogenic activity

**Fig. 2:**
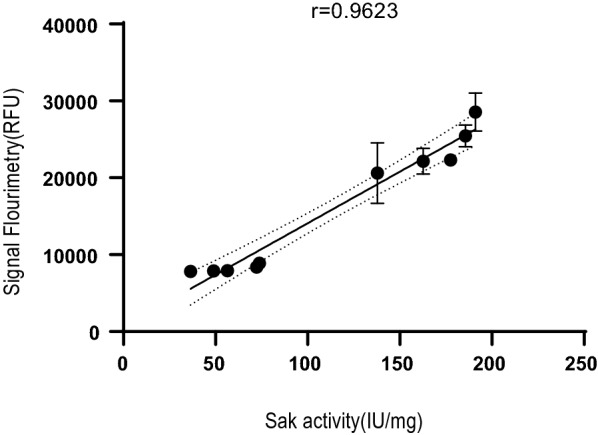
Pearson correlation study between SAK chromogenic activity and fluorescence signals in dicistronic SILEX system. Data are represented with a 95% confidence interval. Data are represented as Mean ± SD from three independent experiments

To investigate the correlation between fluorescence signals and SAK chromogenic activity, 50 clones were first screened by fluorimetry 6 h after inoculation (Additional file 7: Table S3). The lowest and highest fluorescent signals respectively were 13,459 ± 556.5 and 36,306 ± 872.55 RFU. Ten clones with maximum and minimum fluorescent signals were selected for the enzyme activity assay. The Pearson correlation was calculated between fluorescence signals and SAK activity. As shown in Fig. 2, as the fluorescent signal increases, the proteolytic activity of SAK also increases. There was a high positive correlation between the fluorescence signals and SAK activity (Pearson correlation coefficient = 0.9623).

### The SAK expression and plasmid stability of dicistronic SILEX system

**Fig. 3:**
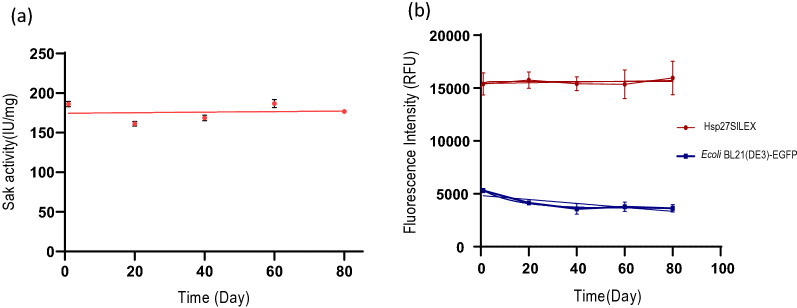
The expression stability of dicistronic SILEX system within 80 days after four freeze–thaw cycles. **a** The SAK chromogenic activity and **b** fluorescent signals. The intensity of fluorescent signals in *E. coli* BL21 (DE3) transformed with pET28a-*sak-rbs-egfp* was measured as the negative control to evaluate the basal expression of eGFP. Data are represented as Mean ± SD from three independent measurements

The expression stability of dicistronic SILEX system was assayed by monitoring the SAK activity and the intensity of fluorescent signals within 80 days after four freeze–thaw cycles. The results demonstrated that the expression level for both proteins was maintained even after 80 days without a noticeable reduction (Fig. 3). In addition, the colony PCR confirmed the plasmid stability of dicistronic SILEX system after 500 days of subculturing by amplifying *hsp27* and *sak rbs egfp* genes (Additional file 8: Fig. S5).

## Discussion

*Escherichia coli* is one of the most common hosts for the production of recombinant proteins; however, the identification of high expressing clones, particularly at lab-scale, is labor-intensive and time-consuming and is often based on traditional methods, namely SDS-PAGE [2, 5]. To date, several high-throughput platforms have been developed to screen the protein expression, most of which focus on automation using robots and qualitative/semi-quantitative screening. These high-throughput technologies allow researchers to screen a large number of clones and remarkably shorten the timeline of protein production at large scale [16]. There are several commercial robotic platforms, such as Equator GX8 Dispenser, MicroSys, and sciFLEXARRAYER dispenser for genomic and proteomic applications, including protein array, antibody array, peptide binding assay, cell-based assay, protein crystallization, and protein expression. These technologies can rapidly transform bacteria, pick colonies based on qualitative or semi-quantitative criteria, screen the protein expression, or even purify the expressed proteins in biological procedures with little human labor. Nonetheless, these platforms are too expensive for most laboratories and need efficient specialists for caring and maintenance of equipments, making them unaffordable in the practice. Hence, if a protein production process does not have a sufficient number of samples to prove this level of cost, it is better to apply a manual method in parallel [17]. However, these manual methods analyze the protein expression within several weeks at a high cost. To resolve such problems, in the current work, we developed a manual method screening a large number of clones just 6 h after the inoculation of newly transformed bacteria to the medium. The method benefits from the following advantages: (i) screening capability in special conditions, such as different temperatures, various media, and variant or fusion expression; (ii) less batch to batch variability; (iii) better handling due to micro-scale; (iv) less error-prone by reducing the steps; (v) screening without employing expensive equipment [2]; (vi) more cost-effective than other screening methods owing to the removal of inducer; (vii) selection in a short time due to the elimination of colony culturing step on LB agar plate, the removal of preculture/induction steps, and the SDS-PAGE technique; (viii) simplicity; (ix) potential capability for automation and use at large scale; (x) obtaining stable high expressing clones due to Hsp27 co-expression; and most importantly, (xi) quantitative screening. Numerous attempts have been previously made to develop a simple and rapid screening method. For example, in a study, the protein expression was monitored by the fusion of photoactive yellow protein to the target protein and the protein concentration and purity were quantified employing a spectrometer within a few minutes [18]. The method, however, needs the addition of a precursor to develop the color that imposes more cost to the system. To this end, herein, we applied an eGFP that does not need any exogenous substrates or cofactors for detection. The attempts by Vincentelli illustrated that the level of soluble protein can be measured in one 96 well-microplate by SDS-PAGE or four 96 well-microplates by dot blot and SDS-PAGE per week. The number of cultures can also be increased up to 1152 (12 × 96 well-microplates) in parallel over 1 week if the process is performed by semi-automated methods, such as liquid handling robots, dot blot, and high-throughput electrophoresis [2]. While in our facilitated protocol, the newly transformed bacteria can be directly inoculated to the medium and screened by fluorescent signals in 96-well microplates. In another study, the intensity of fluorescent signals was measured to analyze the soluble and insoluble proteins. This approach has the following steps: (1) plating different dilutions of transformants on agar plates; (2) cultivation of single colonies in 96-deep-well microplates; (3) induction of expression by adding IPTG; (4) measuring the fluorescent signals by a fluorimeter; (5) and finally, analyzing the soluble and insoluble fractions with SDS-PAGE and western blot [19]. In our previous study, we stated that autoinduction by Hsp27 is a convenient way to produce recombinant proteins without IPTG addition [20]. The developed autoinducible system does not need optical density monitoring, therefore reducing the operator intervention, which is preferable for high-throughput purposes. Additionally, autoinducible systems decrease the cost of production and host cytotoxicity by eliminating the inducer. It is also recommended that the screening protocols must be established at small scales (1–4 ml) to achieve high-throughput capability [2, 16]. In this regard, the current robotic platforms reduce the culture volume by replacing shaker flasks with microplates [21, 22]. Similarly, our screening protocol was optimized at microscale [4]. The correlation analysis of fluorescent signals and enzyme activity revealed that there is a positive relationship between the expression of eGFP and model protein. This is in agreement with previous studies because the transcription of genes was regulated by one promoter in dicistronic vectors [10, 12].

Surprisingly, the designed system resulted in obtaining stable colonies as the high expressing clones kept the initial expression level even after several cycles of freeze–thaw for 80 days. Moreover, our data confirmed the plasmid stability of clones for 500 days. These stabilities can be attributed to the presence of Hsp27 increasing naturally under stress conditions, such as hypoxia, extreme pH or temperatures, and the presence of antibiotics or toxins. The simultaneous expression of heat shock protein with the target protein can rapidly prevent instability and decrease the stress induced by repeated freeze–thaw cycles [23]. This phenomenon is also in agreement with our previous study on SILEX systems [20]. Moreover, since heat shock proteins also act as chaperones, the use of bacteria- or human-originated heat shock proteins in these systems can offer better folding of the complex proteins that need further investigations [24–28].

## Conclusion

As shown in the present work, the combination of dicistronic and autoinducible systems could be employed for developing a rapid and facilitated procedure to screen high-expressing *E. coli* colonies. The developed protocol can be applied as a simple screening method in laboratories without any special equipment owing to the removal of preculture step to save time and cost for the detection of expression. There is also no need for adding an external inducer and elimination of induction step. Simultaneous screening of a large number of clones in a short time, good expression stability after several freeze-thawing cycles, a high correlation between the expression of two genes, and most importantly, quantitative measurement of protein expression at different clones were also among the important advantages of this system. Moreover, the recommended protocol is fully compatible with automation platforms for industrial purposes.

## Materials and methods

### *E. coli* strains, plasmids, and growth conditions

Top10 and DH5-ɑ *E. coli* strains were applied for plasmid propagation and BL21 (DE3) was used for the expression of SAK and eGFP. All the strains were received from the national cell bank of Iran (Pasteur Institute of Iran). The double-transformed bacteria were grown aerobically in Luria Bertani (LB) broth or agar plate supplemented with the appropriate antibiotics (35 µg/ml kanamycin and 100 µg/ml ampicillin) at 37 °C.

### Design of dicistronic SILEX system

The nucleotide sequences encoding Hsp27 (P04792), staphylokinase (*sak*, p68802), and an eGFP protein (peGFP plasmid, Addgene, USA) were optimized and synthesized by Biomatik Canadian Company. The expression cassette containing *sak* and *egfp* was cloned into pET28a within XbaI and XhoI restriction sites. The *hsp27* gene was cloned into pET21a vector within NdeI and XhoI restriction sites. The schematic representations of constructs and the system are depicted in (Additional file 9: Fig. S6).

### Design of screening protocol

#### Optimization of facilitated protocol for colony screening

The following steps were considered in the optimization of facilitated screening protocol through a dicistronic expression system containing eGFP and SAK protein autoinduced by a system comprising Hsp27 as an autoinducer: (1) optimization of double transformation protocol in terms of optical density of bacterial culture (OD_600nm_ of 0.3, 0.4, 0.45, and 0.5) for entering to the competent stage, the use of different plasmid amounts (200, 300, 400, and 500 ng) to obtain a specific number of transformants (~ 100 transformants) for transferring a clone in each well of 96-microplate; (2) optimization of incubation time in cold CaCl_2_ solution (15 min to 18 h) for competent cell preparation for obtaining ~ 100 double-transformed bacteria; (3) investigation of different volumes of transformation solution (2, 10, 12, 20, and 25 µl) for obtaining a single double-transformed colony in each well of 96-well microplates and removing the overnight culture step for protein expression; and (4) determination of optimal time to record fluorescence signals on account of eGFP expression (minimum time required for more accurate screening of protein expression). In this test, fluorescent signals were measured for 10 different clones of dicistronic SILEX system within 6 h with an interval of 1 h. Moreover, the fluorescence sensitivity of the device was adjusted to the specified values (60, 70, and 80 RFU) and eGFP expression signal was measured for 26 individual clones 6 h after inoculation. Because if the sensitivity of the fluorimeter is more than a threshold, eGFP expression is out of the detection range (overflow) and if is less than a threshold, it interferes with the background signals. Moreover, the precision, linearity, the limit of detection (LOD), and the limit of quantification (LOQ) were measured for the facilitated protocol. The precision was determined by measuring relative standard deviation (RSD). The detection and quantification limits were determined for 10 samples as a mean of sample blank value plus three and 10 times of its standard deviation, respectively. The LOD can be expressed as the smallest amount or concentration of eGFP that can be reliably detected or distinguished from the background signal of fluorimeter device and blank sample (the suspension of *E. coli* culture without pET28a –*sak rbs egfp* plasmid)*.* The limit of quantifying a specific analytical procedure can be determined as the smallest amount or concentration of eGFP that can be measured quantitatively with acceptable precision and accuracy [15].

### Expression analysis of SAK with SDS-PAGE, western blot, and purification

The expression of SAK protein was analyzed by use of SDS-PAGE, western blot, and affinity purification. Briefly, a clone was inoculated into a 5-ml LB medium containing 100 µg/ml of ampicillin and 35 of μg/ml kanamycin and incubated for 16 h in a shaker incubator at 37 °C/170 rpm. Afterwards, the 5-ml and 1-L LB cultures were respectively inoculated by 100 µl and 20 ml pre-culture and incubated at 37 °C/250 rpm overnight. The SAK expression was analyzed through the use of 15% gel SDS-PAGE and Coomassie blue staining method and confirmed with western blot technique. In brief, the sample was transferred on nitrocellulose membrane (Sigma-Aldrich, USA) and blocked with a TBST buffer (Tris-buffered saline (Tris 20 mM with pH: 7.5, NaCl 150 mM), 0.1% Tween 20, and 3% bovine serum albumin) for 2 h. The membrane was then incubated in a buffer containing anti-HisTag antibody (Sigma- Aldrich, USA) with a dilution of 1:2000 at 4 °C for 16 h. Finally, the protein band was visualized by adding DAB (3, 3'-Diaminobenzidine) and H_2_O_2_. To purify SAK, the pellet was resuspended in the lysis buffer (300 mM NaCl, 50 mM NaH_2_PO_4_.2H_2_O, 1 mg/ml lysozyme, pH 8) and lysed by sonication on ice for 20 min. The lysate was then centrifuged at 14,000*g* for 25 min at 4 °C and the supernatant was loaded on an equilibrated Ni–NTA resin (Qiagen, USA) for 1 h at 4 °C according to the manufacturer's protocol. The resin was washed three times with the wash buffer (300 mM NaCl, 50 mM NaH_2_PO_4_.2H_2_O, 20 mM imidazole, pH 8). Lastly, the protein was eluted by a buffer containing 300 mM NaCl, 50 mM NaH_2_PO_4_.2H_2_O, and 250 mM imidazole (adjusted to pH 8).

### Enzyme activity measurement

The SAK activity was determined qualitatively and quantitatively respectively applying radial caseinolytic assay and chromogenic methods. In the radial caseinolytic assay, a double-transformed clone was inoculated into a 5-ml LB medium supplemented with 35 μg/ml of kanamycin and 100 µg/ml of ampicillin and incubated in a shaker incubator at 37 °C/170 rpm overnight. Afterwards, 100 µl of the suspension was added to a 5-ml LB medium and incubated at 37 °C/250 rpm for 16 h. The culture was then centrifuged at 4 °C/4000*g* and the pellet was washed twice with cold PBS. The pellet was resuspended in the lysis buffer (50 mM NaH_2_PO_4_.2H_2_O, 300 mM NaCl, 1 mg/ml lysozyme, pH 7.5). The lysate was then poured into the holes on 5% skim milk agar plates (0.05 g/ml skim milk, 0.04 g/ml LB agar, 50 mM Tris–HCl, 0.15 M NaCl, pH 7.5) supplemented with 8 µl of Glu-plasminogen (142.85 µM) in triplicate and incubated at 37 °C for 18 h. The reference SAK (Prospect, 50,000 IU/mg) with and without Glu-plasminogen were utilized as positive and negative controls, respectively [29]. The clear zone diameters were then determined by ImageJ software (https://imagej.nih.gov/ij/index.html).

In the chromogenic method, nine precultures of the double transformed colonies were separately inoculated into a 5-ml LB medium and incubated for 16 h at 37 °C/170 rpm. Afterwards, 10 µl of bacterial suspensions (OD_600nm_ =  ~ 2.5) were added into a well of 96-microplate containing 190 µl of LB broth supplemented with 35 μg/ml of kanamycin and 100 µg/ml of ampicillin in triplicate. The plate was incubated at 37 °C/90 rpm for 6 h. Finally, the activity of SAK in the clones was determined according to an activity-absorbance standard curve. To obtain the standard curve, serial dilutions of the reference SAK was prepared and 8 of µl human Glu-plasminogen solution (142.85 µM, Invitrogen) was mixed with 30 µl of SAK standard solutions (0.04, 0.09, 0.39, 0.75, 1.56, 6.25, and 13.5 µM, Prospect) in triplicates and the plate was incubated for 25 min at 37 °C [30]. Afterwards, 7 µl of H-D-Val-Leu-Lys-paranitroanilide as plasminogen substrate (50 mg/ml, S2251, Sigma-Aldrich) was added to the wells and the plate was incubated for 30 min at 37 °C. Finally, the absorbance was measured at a wavelength of 405 nm.

### Correlation study between fluorescence signals and SAK activity

To investigate the association between fluorescence signals and SAK activity, 50 precultures of double-transformed colonies were separately inoculated into a 5-ml LB medium and incubated for 16 h at 37 °C/170 rpm. Next, 10 µl of bacterial suspensions (OD_600nm_ =  ~ 2.5) were added to a 96-microplate containing 190 µl of LB broth supplemented with 35 μg/ml of kanamycin and 100 µg/ml of ampicillin in triplicate. The plate was incubated at 37 °C/90 rpm for 6 h. The fluorescent signals were finally measured using a fluorimeter (485 nm excitation and 528 nm emission, BioTek, USA). Then, 10 clones with high and low fluorescence signals (five clones for each group) were selected, inoculated individually into a 5 ml LB medium, and incubated for 16 h at 37 °C/170 rpm. Subsequently, 10 µl of bacterial precultures was transferred into a 96 wells-microplate containing 190 µl of LB broth and the selective antibiotics in triplicate and the plate was incubated for 6 h at 37 °C/90 rpm. The fluorescent signals were finally measured using fluorimetry (485 nm excitation and 528 nm emission, BioTek, USA). For enzyme activity measurement, 8 µl of Glu-plasminogen (142.85 µM) was added to 200 µl of bacterial suspensions and the mixtures were incubated for 30 min at 25 °C in triplicate. Following incubation, 7 µl of synthetic substrate S2251 (50 mg/ml) was added to the mixtures and the absorbance was measured at a wavelength of 405 nm with a spectrophotometer (Epoch, BioTek, USA).

### Expression and plasmid stability of dicistronic system

The expression stability was measured after four freeze-thawing cycles at − 70 °C for three clones. In brief, the bacteria were double-transformed with the plasmids and the fluorescent intensity and the enzyme activity were measured as mentioned before. The double-transformed bacteria were stored at − 70 °C and after 20 days, 100 µl of glycerol stocks were inoculated into a 5-ml LB broth medium supplemented with the selective antibiotics and incubated for 16 h at 37 °C/170 rpm. Afterwards, 100 µl of precultures were inoculated to a 5-ml LB broth and incubated for 16 h at 37 °C/250 rpm and the fluorescent intensity and the enzyme activity of clones were measured again. The freeze-thawing cycle was repeated four times within 80 days. The plasmid stability was also investigated with colony PCR after 500 days of subculturing on LB agar plates with 10 days intervals. Universal T7-promoter and T7-terminator primers were utilized to amplify the genes (*hsp27* and *sak rbs egfp*). Briefly, the pellet obtained from a newly cultured clone was lysed by boiling and added to a PCR reaction containing 2 µl of forward and reverse primers (20 µM), 10 µl of *Taq* DNA Polymerase Master Mix-Amicon, and ddH_2_O up to 50 µl. The PCR program was as follows: 95 °C for 5 min (one cycle); 95 °C for 30 s; 49 °C for 30 s; and 72 °C for 30 s (35 cycles); and a final extension of 72 °C for 10 min.

### The screening protocol

**Fig. 4:**
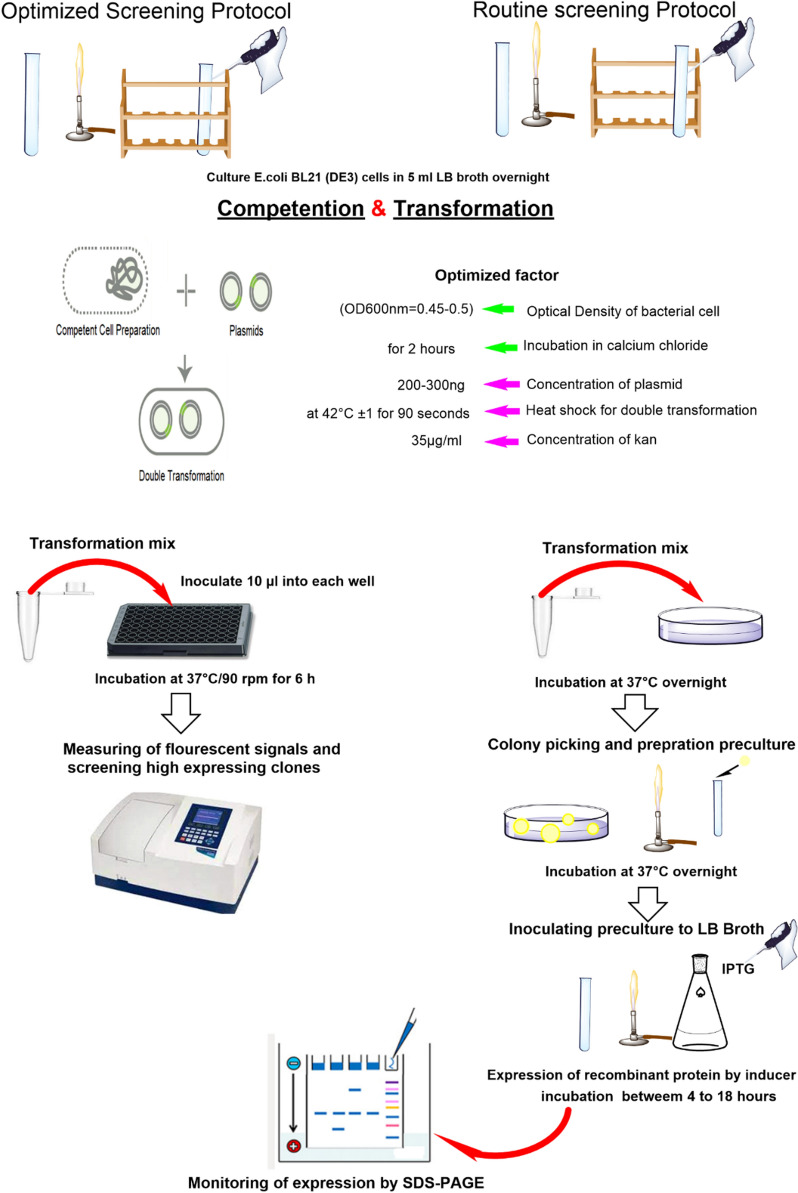
The schematic representation of the developed screening protocol

The developed screening protocol is as follows. The schematic representation of the screening protocol is also portrayed in Fig. 4.

1. Inoculate 200 µl of seed culture into a 5-ml LB broth;
2. Incubate the culture at 37 °C/250 rpm up to OD_600nm_ ~ 0.5;
3. Centrifuge the culture at 4 °C/4000*g* for 5 min and resuspend the pellet in 1200 μl cold CaCl_2_ (0.1 M);
4. Incubate the bacterial suspension at 4 °C for 15 min;
5. Centrifuge the bacterial suspension at 4 °C/4000*g* for 5 min;
6. Add 1200 μl of cold CaCl_2_ (0.1 M) to the pellet, resuspend it, and incubate the bacterial suspension at 4 °C for 2 h;
7. Centrifuge the bacterial suspension at 4 °C/4000*g* for 5 min;
8. Resuspend the pellet in 300 μl of cold CaCl_2_ (0.1 M) to obtain the competent cells;
9. Add pET21a-*hsp27* and pET28a-*sak-rbs-egfp* plasmids (200–300 ng) to 100 μl of the competent cells;
10. Incubate the mixture at 4 °C for 30 min and then for 90 s at 42 °C;
11. Transfer the mixture on ice for 3 min immediately;
12. Add 1 ml of LB broth to the double-transformed bacteria and incubate for 1 h at 37 °C/220 rpm;
13. Inoculate 10 µl of the transformed cells into the wells of a 96-microplate containing 190 µl of LB broth supplemented with selective antibiotics (herein, 35 µg/ml kanamycin and 100 µg/ml ampicillin);
14. Incubate the microplate at 37 °C/90 rpm for 6 h;
15. Screen high expressing colonies using fluorimetry (485 nm excitation and 528 nm emission).

### Statistical analysis and additional materials and methods

All data were statistically analyzed with SPSS software version 25. Plotting and curve fitting was performed using Prism software version 8. Materials and methods for supplementary files were provided in Additional file 10. 

## Supplementary Information

**Additional file 1: Table S1.** Optimization of plasmid amount and OD_600nm_ of bacterial cultures at the double-transformation process to obtain approximately 100 colones.

**Additional file 2: Table S2.** Optimization of transferring different volumes of transformation suspension to obtain a single clone in each well of 96-well microplates. Data are represented from three independent experiments.

**Additional file 3: Fig. S1.** Time determination for fluorimetry.

**Additional file 4: Fig. S2.** Fluorescence sensitivity measurements. Fluorescent signals were measured for 26 clones at the sensitivities of 60, 70, and 80. The only signals with a relative fluorescence unit (RFU) of < 100,000 were pointed out in the figure because the others were out of detection (overflow). The sensitivity of 60 was chosen because the fluorescent signals for all the clones were measurable while five and 11 clones were out of detection for the sensitivity of 70 and 80, respectively.

**Additional file 5: Fig. S3.** SAK activity measurement on 5% skim milk-agar plate in presence of plasminogen: (i) wells 1, 2, and 3, reference SAK without adding plasminogen (as a negative control, triplicate); (ii) wells 4, 5, and 6, reference SAK with adding plasminogen (as a positive control, triplicate); (iii) wells 7, 8, and 9, the expressed SAK in dicistronic SILEX system at three different clones.

**Additional file 6: Fig. S4.** The standard curve for the SAK activity. Data are represented as Mean ± SD from three independent measurements. There is a linear relationship between the SAK activity (IU/mg) and the absorbance at a wavelength of 405 nm.

**Additional file 7: Table S3.** Screening of 50 clones by fluorimetry 6 h after inoculation in a 96-well microplate. Ten clones with maximum and minimum fluorescent signals were selected for the enzyme activity assay.

**Additional file 8: Fig. S5.** The plasmid stability in dicistronic SILEX system after 500 days of subculturing. The PCR amplified bands were visualized on 2% gel agarose. Lane1, *E. coli* BL21(DE3) without plasmids (negative control); Lane 2, DNA Ladder; Lane 3, a newly double-transformed *E. coli* BL21(DE3) containing pET21a-*hsp70* (1927 bp for *hsp70*) and pET28a-*sak*-*rbs*-*egfp* (1024 bp for *egfp*) as a positive control; Lane 4, dicistronic SILEX system containing pET21a-*hsp27* (619 bp for *hsp27*) and pET28a-*sak*-*rbs*-*egfp* (1024 bp for *egfp*) after 500 days of subculturing.

**Additional file 9: Fig. S6.** The schematic representation of (a) the designed constructs and (b) dicistronic SILEX system.

**Additional file 10.** Additional materials and methods for additional files 1 to 9.

## Data Availability

All data generated or analyzed during this study are included in the published article and supplementary file and or are available from the corresponding author on reasonable request.

## Abbreviations

eGFP: Enhanced green fluorescent protein
SAK: Staphylokinase
LOD: Limit of detection
LOQ: Limit of quantification
FACS: Fluorescence-activated cell sorting
RBS: Ribosome-binding sites
SILEX: Self-induced expression system
RFU: Relative fluorescent unit
RSD: Relative standard deviation
TBST: Tris-buffered saline
