# Supplementary material for: Rapid screening of high expressing Escherichia coli colonies using a novel dicistronic-autoinducible system
Source: Microb Cell Fact. 2021 Dec 11;20:223. doi: 10.1186/s12934-021-01711-2 (PMC8666062; doi:10.1186/s12934-021-01711-2)
Supplement: Supplementary file 1 — Additional file 1: Table S1. Optimization of plasmid amount and OD600nm of bacterial cultures at the double-transformation process to obtain approximately 100 colones. [file 12934_2021_1711_MOESM1_ESM.docx]

**Additional file 1. Table S1**. Optimization of plasmid amount and OD_600nm_ of bacterial cultures at the double-transformation process to obtain approximately 100 colones.

| **Optimization Criteria** | **Plasmid (ng)** | | | | **OD_600nm_ of Bacterial Cultures** | | | |
| --- | --- | --- | --- | --- | --- | --- | --- | --- |
|  | 200 | 300 | 400 | 500 | 0.3 | 0.4 | 0.45 | 0.5 |
| **Number of Double-Transformants** | 94.33 ± 3.29 | 115.67 ± 3.29 | 200-400 | 300-600 | 25.33 ± 2.05 | 55.33 ± 4.10 | 97.66 ± 2.05 | 107.33 ± 2.49 |
| **RSD (%)*** | 3.49 | 2.85 | - | - | 8.11 | 7.42 | 2.10 | 2.32 |

* Relative Standard Deviation
