# Supplementary material for: Rapid screening of high expressing Escherichia coli colonies using a novel dicistronic-autoinducible system
Source: Microb Cell Fact. 2021 Dec 11;20:223. doi: 10.1186/s12934-021-01711-2 (PMC8666062; doi:10.1186/s12934-021-01711-2)
Supplement: Supplementary file 2 — Additional file 2: Table S2. Optimization of transferring different volumes of transformation suspension to obtain a single clone in each well of 96-well microplates. Data are represented from three independent experiments. [file 12934_2021_1711_MOESM2_ESM.docx]

**Additional file 2. Table S2**. Optimization of transferring different volumes of transformation suspension to obtain a single clone in each well of 96-well microplate. Data are represented from three independent experiments.

| **Transferring Volume (µl)** | 2 | 10 | 12 | 20 | 25 |
| --- | --- | --- | --- | --- | --- |
| **Number of clones** | 0 ± 0.00 | 1 ± 0.00 | 1 ± 0.00 | 1.33 ± 0.47 | 1.66 ± 0.47 |

Data are represented from three independent experiments.
