# Supplementary material for: Rapid screening of high expressing Escherichia coli colonies using a novel dicistronic-autoinducible system
Source: Microb Cell Fact. 2021 Dec 11;20:223. doi: 10.1186/s12934-021-01711-2 (PMC8666062; doi:10.1186/s12934-021-01711-2)
Supplement: Supplementary file 3 — Additional file 3: Fig. S1. Time determination for fluorimetry. [file 12934_2021_1711_MOESM3_ESM.docx]

**Additional file 3. Fig. S1**. Time determination for fluorimetry.
