# Supplementary material for: Rapid screening of high expressing Escherichia coli colonies using a novel dicistronic-autoinducible system
Source: Microb Cell Fact. 2021 Dec 11;20:223. doi: 10.1186/s12934-021-01711-2 (PMC8666062; doi:10.1186/s12934-021-01711-2)
Supplement: Supplementary file 4 — Additional file 4: Fig. S2. Fluorescence sensitivity measurements. Fluorescent signals were measured for 26 clones at the sensitivities of 60, 70, and 80. The only signals with a relative fluorescence unit (RFU) of < 100,000 were pointed out in the figure because the others were out of detection (overflow). The sensitivity of 60 was chosen because the fluorescent signals for all the clones were measurable while five and 11 clones were out of detection for the sensitivity of 70 and 80, respectively. [file 12934_2021_1711_MOESM4_ESM.docx]

**Additional file 4. Fig. S2**. Fluorescence sensitivity measurements. Fluorescent signals were measured for 26 clones at sensitivities of 60, 70, and 80. The only signals had a relative fluorescence unit (RFU) of <100000 were pointed out in the figure because the others were out of detection (overflow). The sensitivity of 60 was chosen because the fluorescent signals for all clones were measurable. While 5 and 11 clones were out of detection for the sensitivity of 70 and 80, respectively.
