# Supplementary material for: Rapid screening of high expressing Escherichia coli colonies using a novel dicistronic-autoinducible system
Source: Microb Cell Fact. 2021 Dec 11;20:223. doi: 10.1186/s12934-021-01711-2 (PMC8666062; doi:10.1186/s12934-021-01711-2)
Supplement: Supplementary file 5 — Additional file 5: Fig. S3. SAK activity measurement on 5% skim milk-agar plate in presence of plasminogen: (i) wells 1, 2, and 3, reference SAK without adding plasminogen (as a negative control, triplicate); (ii) wells 4, 5, and 6, reference SAK with adding plasminogen (as a positive control, triplicate); (iii) wells 7, 8, and 9, the expressed SAK in dicistronic SILEX system at three different clones. [file 12934_2021_1711_MOESM5_ESM.docx]

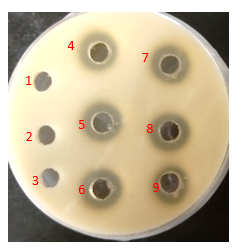


**Additional file 5. Fig. S3**. SAK activity measurements on 5% skim milk-agar plate in the presence of plasminogen: (i) Wells 1, 2, and 3; Reference SAK without adding plasminogen (As a negative control, triplicate), (ii) wells 4, 5, and 6; Reference SAK with adding plasminogen (As a positive control, triplicate), (iii) wells 7, 8, and 9; The expressed SAK in dicistronic SILEX system at three different clones.
