# Supplementary material for: Rapid screening of high expressing Escherichia coli colonies using a novel dicistronic-autoinducible system
Source: Microb Cell Fact. 2021 Dec 11;20:223. doi: 10.1186/s12934-021-01711-2 (PMC8666062; doi:10.1186/s12934-021-01711-2)
Supplement: Supplementary file 6 — Additional file 6: Fig. S4. The standard curve for the SAK activity. Data are represented as Mean ± SD from three independent measurements. There is a linear relationship between the SAK activity (IU/mg) and the absorbance at a wavelength of 405 nm. [file 12934_2021_1711_MOESM6_ESM.docx]

**Additional file 6. Fig. S4**. The standard curve for SAK activity. Data are represented as Mean ± SD from three independent measurements. There is a linear relationship between the SAK activity (IU/mg) and the absorbance at a wavelength of 405 nm.
