# Supplementary material for: Rapid screening of high expressing Escherichia coli colonies using a novel dicistronic-autoinducible system
Source: Microb Cell Fact. 2021 Dec 11;20:223. doi: 10.1186/s12934-021-01711-2 (PMC8666062; doi:10.1186/s12934-021-01711-2)
Supplement: Supplementary file 7 — Additional file 7: Table S3. Screening of 50 clones by fluorimetry 6 h after inoculation in a 96-well microplate. Ten clones with maximum and minimum fluorescent signals were selected for the enzyme activity assay. [file 12934_2021_1711_MOESM7_ESM.docx]

**Additional file 7. Table S3**. Screening of 50 clones by fluorimetry after 6 h inoculation in a 96-well microplate. Ten clones with maximum and minimum fluorescent signals were selected for the enzyme activity assay.

| **Clone No.** | **Fluorescent Intensity (RFU)** | **Clone No.** | **Fluorescent Intensity (RFU)** |
| --- | --- | --- | --- |
| 1 | 26949 ± 660.56 | 26 | 29292 ± 432.30 |
| 2 | 30145 ± 1072.63 | 27 | 26819 ± 70.26 |
| 3 | 28446 ± 762.19 | 28 | 28179 ± 579.98 |
| 4 | 24673 ± 1092.47 | 29 | 27180 ± 295.74 |
| 5 | 28344 ± 1011.9 | 30 | 29424 ± 31.36 |
| 6 | 29297 ± 80.58 | 31 | 29633 ± 141.72 |
| 7 | 26134 ± 16.02 | 32 | 31664 ± 99.64 |
| 8 | 25994 ± 551.00 | **33** | **36306 ± 872.55** |
| 9 | 29232 ± 850.72 | 34 | 32498 ± 74.63 |
| 10 | 29398 ± 109.94 | **35** | **35323 ± 1307.24** |
| 11 | 29030 ± 992.44 | 36 | 30700 ± 473.59 |
| 12 | 29451 ± 222.30 | 37 | 29415 ± 21.03 |
| 13 | 28404 ± 195.70 | 38 | 30260 ± 473.59 |
| 14 | 30737 ± 749.88 | **39** | **32498 ± 21.03** |
| **15** | **35955 ± 685.97** | **40** | **35323 ± 1307.24** |
| 16 | 30126 ± 239.37 | 41 | 30700 ± 1634.08 |
| 17 | 33878 ± 45.65 | 42 | 29415 ± 21.03 |
| 18 | 30337 ± 620.87 | 43 | 30260 ± 18.65 |
| 19 | 29280 ± 271.53 | 44 | 28562 ± 1634.08 |
| 20 | 30059 ± 508.92 | **45** | **18271 ± 1380.67** |
| 21 | 27111 ± 63.51 | **46** | **15521 ± 275.06** |
| 22 | 31724 ± 147.67 | **47** | **18687 ± 398.65** |
| 23 | 27097 ± 1026.97 | 48 | 22326 ± 3601.49 |
| 24 | 28080 ± 45.25 | **49** | **22138 ± 1687.41** |
| 25 | 28269 ± 532.65 | **50** | **13459 ± 556.5** |

Bolded rows indicate 10 clones with minimum and maximum expressions.
