# Supplementary material for: Rapid screening of high expressing Escherichia coli colonies using a novel dicistronic-autoinducible system
Source: Microb Cell Fact. 2021 Dec 11;20:223. doi: 10.1186/s12934-021-01711-2 (PMC8666062; doi:10.1186/s12934-021-01711-2)
Supplement: Supplementary file 8 — Additional file 8: Fig. S5. The plasmid stability in dicistronic SILEX system after 500 days of subculturing. The PCR amplified bands were visualized on 2% gel agarose. Lane1, E. coli BL21(DE3) without plasmids (negative control); Lane 2, DNA Ladder; Lane 3, a newly double-transformed E. coli BL21(DE3) containing pET21a-hsp70 (1927 bp for hsp70) and pET28a-sak-rbs-egfp (1024 bp for egfp) as a positive control; Lane 4, dicistronic SILEX system containing pET21a-hsp27 (619 bp for hsp27) and pET28a-sak-rbs-egfp (1024 bp for egfp) after 500 days of subculturing. [file 12934_2021_1711_MOESM8_ESM.docx]

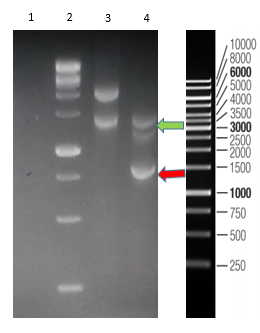


**Additional file 8. Fig. S5**. The plasmid stability in dicistronic SILEX system after 500 days of subculturing. The PCR amplified bands were visualized on 2% gel agarose. Lane1; *E.coli* BL21(DE3) without plasmids (negative control), Lane 2; DNA Ladder, Lane 3; a newly double-transformed *E.coli* BL21(DE3) containing pET21a-*hsp70* (1927 bp for *hsp70*) and pET28a-*sak*-*rbs*-*egfp* (1024 bp for *egfp*) as a positive control, and Lane 4; Dicistronic SILEX system containing pET21a-*hsp27* (619 bp for *hsp27*) and pET28a-*sak*-*rbs*-*egfp* (1024 bp for *egfp*) after 500 days of subculturing.
