# Supplementary material for: Rapid screening of high expressing Escherichia coli colonies using a novel dicistronic-autoinducible system
Source: Microb Cell Fact. 2021 Dec 11;20:223. doi: 10.1186/s12934-021-01711-2 (PMC8666062; doi:10.1186/s12934-021-01711-2)
Supplement: Supplementary file 9 — Additional file 9: Fig. S6. The schematic representation of (a) the designed constructs and (b) dicistronic SILEX system. [file 12934_2021_1711_MOESM9_ESM.docx]

(a)


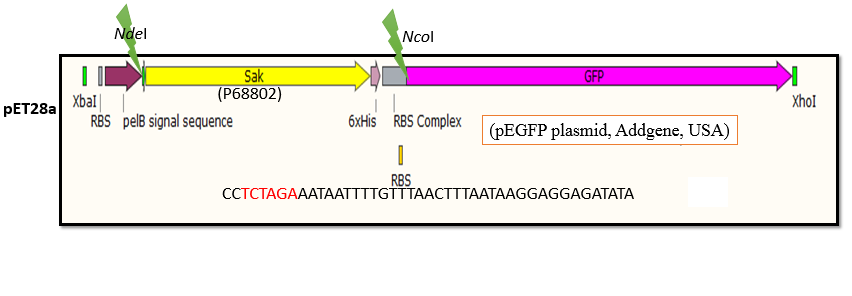


(b)


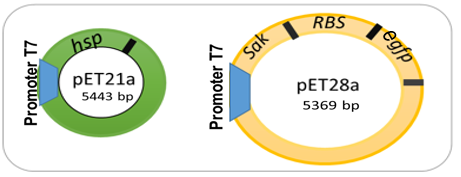


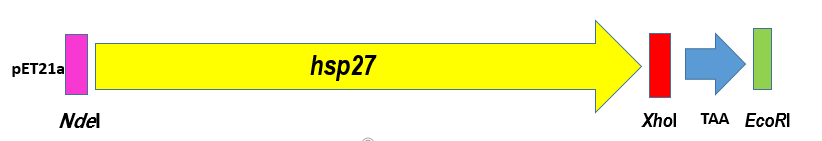


**Additional file 9. Fig. S6**. The schematic representation of (a) the designed constructs and (b) dicistronic SILEX system.
