# Supplementary material for: Rapid screening of high expressing Escherichia coli colonies using a novel dicistronic-autoinducible system
Source: Microb Cell Fact. 2021 Dec 11;20:223. doi: 10.1186/s12934-021-01711-2 (PMC8666062; doi:10.1186/s12934-021-01711-2)
Supplement: Supplementary file 10 — Additional file 10. Additional materials and methods for additional files 1 to 9. [file 12934_2021_1711_MOESM10_ESM.docx]

**Materials and Methods Additional File**

**Rapid Screening of High Expressing *Escherichia coli* Colonies using a Novel Dicistronic-Autoinducible System**

Fatemeh Sadat Shariati^1^, Dariush Norouzian^1^, Vahideh Valizadeh^1^, Reza Ahangari Cohan^[[1]](#footnote-1)*^, Malihe Keramati ^1*^

^1^Department of Nanobiotechnology, New Technologies Research Group, Pasteur Institute of Iran, Tehran, Iran

**Methods for additional file 1**

In double transformation process, different amounts of plasmids with a ratio of 1:1 and different optical densities of bacterial cultures were tested to obtain a specific number of double-transformants (~100). The number of double-transformants in each step was determined by plating on the LB agar plates containing both selective antibiotics followed by colony counting.

**Methods for additional file 2**

For removing the spreading step of newly double-transformed cells on LB agar plate and the overnight culture step for protein expression, direct inoculation of transformation solution to each well of 96-microplate was proposed. For this reason, the inoculation was investigated at different volumes (2, 10, 12, 20, and 25 µl) of the newly transformed suspension on LB agar plates containing both selective antibiotics to obtain a single clone in each well of 96-microplate. The number of clones was determined by plating on the LB agar plates containing both selective antibiotics followed by colony counting.

**Methods for additional file 3**

The optimum time record of fluorescence signals (eGFP expression) was investigated to obtain the minimum time required for a more accurate screening of protein expression. In this experiment, the fluorescent signals were measured for 10 different clones for 6 h at 1 hour intervals.

**Methods for additional file 4**

Fluorescent signals were measured for different 26 clones of dicistronic SILEX system at three sensitivities of 60, 70, and 80 at a volume of 200 µl to select the best sensitivity of the fluorimeter. Because, if the sensitivity of the fluorimeter is more than a threshold, the eGFP expression is out of the detection range (overflow), and if is less than a threshold, it interferes with the background signals.

**Methods for additional file 5**

In the radial caseinolytic assay, a double-transformed clone was inoculated into a 5 mL LB medium supplemented with 35 μg/mL kanamycin and 100 µg/mL ampicillin and incubated in a shaker incubator at 37ºC/170 rpm, overnight. Then, 100 µL suspension was added to 5 mL LB medium and incubated at 37 ºC/250 rpm for 16 h. The culture was centrifuged at 4ºC/4000 g and the pellet was washed twice with cold PBS. The pellet was resuspended in the lysis buffer (50 mM NaH_2_PO_4_.2H_2_O, 300 mM NaCl, and 1 mg/ml lysozyme, pH 7.5). The lysate was then poured into the holes on 5% skim milk agar plates (0.05 g/mL skim milk, 0.04 g/mL LB agar, 50 mM Tris-HCl, 0.15 M NaCl, pH 7.5) supplemented with 8 µl Glu-plasminogen (142.85 µM) in triplicate and incubated at 37 ºC for 18 h. The reference SAK (Prospect, 50000 IU/mg) with or without Glu-plasminogen was used as positive and negative controls, respectively. The clear zone diameters were then determined by ImageJ software (<https://imagej.nih.gov/ij/index.html>).

**Methods for additional file 6**

To obtain the standard curve, serial dilutions of reference SAK was prepared, and 8 µl human Glu-plasminogen solution (142.85 µM, Invitrogen) was mixed with 30 µl SAK standard solutions (0.04, 0.09, 0.39, 0.75, 1.56, 6.25, and 13.5 µM, Prospect) in triplicates and the plate was incubated for 25 min at 37 ºC [30]. Seven microliters of H-D-Val-Leu-Lys-paranitroanilide as plasminogen substrate (50 mg/mL, S2251, Sigma-Aldrich) were then added to the wells and the plate was incubated for 30 min at 37 °C. Finally, the absorbance was measured at a wavelength of 405 nm.

**Methods for additional file 7**

To investigate the correlation between fluorescence signals and SAK activity, 50 precultures of double-transformed colonies were separately inoculated into a 5 mL LB medium and incubated for 16 h at 37ºC/170 rpm. Ten microliters of bacterial suspensions (OD_600nm_ = ~2.5) were then added into a 96-microplate containing 190 µL LB broth supplemented with 35 μg/mL kanamycin and 100 µg/ml ampicillin in triplicate. The plate was incubated at 37ºC/90 rpm for 6 h. The fluorescent signals were finally measured using a fluorimeter (485 nm excitation and 528 nm emission, BioTek, USA). Then, 10 clones with high and low fluorescence signals (five clones for each group) were selected, inoculated individually into a 5 mL LB medium, and incubated for 16 h at 37ºC/170 rpm. Then, 10 µl bacterial precultures were transferred into a 96 wells-microplate containing 190 µl LB broth and the selective antibiotics in triplicate, and the plate was incubated for 6 h at 37°C/90 rpm. The fluorescent signals were finally measured using fluorimetry (485 nm excitation and 528 nm emission, BioTek, USA). For enzyme activity measurement, 8 µl Glu-plasminogen (142.85 µM) was added to 200 µl bacterial suspensions and the mixtures were incubated for 30 min at 25°C in triplicate. After incubation, 7 µl synthetic substrate S2251 (50 mg/ml) was added to the mixtures and the absorbance was measured at a wavelength of 405 nm by a spectrophotometer (Epoch, BioTek, USA).

**Methods for additional file 8**

The plasmid stability was also investigated by colony PCR after 500 days of subculturing on LB agar plates with 10 days’ intervals. Universal T7-promoter and T7-terminator primers were used to amplify the genes (*hsp27* and *sak* *rbs* *egfp*). Briefly, the pellet obtained from a newly cultured clone was lysed by boiling and added to a PCR reaction containing 2 µl forward and reverse primers (20 µM), 10 µl of *Taq* DNA Polymerase Master Mix-Amicon, and ddH_2_O up to 50 µl. The PCR program was as follows; 95 °C for 5 min (1 cycle), 95 °C for 30 sec, 49 °C for 30 sec, and 72 °C for 30 sec (35 cycles), and a final extension of 72 °C for 10 min.

**Methods for additional file 9**

Not applicable.

1. *Corespondences: [cohan_r@pasteur.ac.ir](mailto:cohan_r@pasteur.ac.ir%20), keramati.malihe@gmail.com

   ^1^Department of Nanobiotechnology, New Technologies Research Group, Pasteur Institute of Iran, Tehran, Iran [↑](#footnote-ref-1)
